# THE GLOBAL PRIDE STUDY: LESSONS LEARNED AND NEXT STEPS

**DOI:** 10.1093/geroni/igae098.1088

**Published:** 2024-12-31

**Authors:** Karen Fredriksen-Goldsen, Christi Nelson, Austin Oswald, Hyun-Jun Kim

**Affiliations:** University of Washington, Seattle, Washington, United States; University of Washington, Seattle, Washington, United States; Dalhousie University, Nova Scotia, Canada; University of Washington, Seattle, Washington, United States

## Abstract

As the older adult population becomes increasingly diverse and globalized, new challenges emerge in gerontological research and practice worldwide. This presentation examines sexuality, gender, and aging within a global context, exploring both fortitude and risks, as well as differing social and cultural interpretation of sexuality, gender, and age in relation to research and the development of culturally relevant practices. We will discuss the formation of the research network, involving more than 50 international collaborators. As part of the Global Pride project, we developed and tested a pilot survey, which was distributed by 18 partners and completed across six global regions (N= 3,885). Key processes and lessons learned include: 1. Establishing an international network of researchers to exchange knowledge and experiences in studying sexuality, gender, longevity, and health holistically; 2. Implementing a collaboratively developed survey across six global regions, taking into consideration differing cultural contexts and methodologies; 3. Employing culturally tailored dissemination strategies across diverse regions and settings. The insights gleaned from this research will deepen an awareness of the diversity of lives across the life course and contribute knowledge about the richness, challenges, and heterogeneity of ageing, as well as best practices for the implementation of global research.

